# Feel to Heal: Negative Emotion Differentiation Promotes Medication Adherence in Multiple Sclerosis

**DOI:** 10.3389/fpsyg.2021.687497

**Published:** 2022-01-10

**Authors:** T. H. Stanley Seah, Shaima Almahmoud, Karin G. Coifman

**Affiliations:** Department of Psychological Sciences, Kent State University, Kent, OH, United States

**Keywords:** emotion, emotion differentiation, health behaviors, medication adherence, multiple sclerosis, chronic illness

## Abstract

Multiple Sclerosis (MS) is a debilitating chronic autoimmune disease of the central nervous system that results in lower quality of life. Medication adherence is important for reducing relapse, disease progression, and MS-related symptoms, particularly during the early stages of MS. However, adherence may be impacted by negative emotional states. Therefore, it is important to identify protective factors. Past research suggests that the ability to discriminate between negative emotional states, also known as negative emotion differentiation (NED), may be protective against enactment of maladaptive risk-related behaviors. However, less is known as to how NED may promote adaptive health behaviors such as medication adherence. Utilizing weekly diaries, we investigated whether NED moderates the association between negative affect and medication adherence rates across 58 weeks among patients (*n* = 27) newly diagnosed with MS (following McDonald criteria). Results revealed that NED significantly moderated the relationship between negative affect and medication adherence. Specifically, greater negative affect was associated with lower adherence only for individuals reporting low NED. However, this link disappeared for those reporting moderate to high NED. Building upon past research, our findings suggest that NED may promote adaptive health behaviors and have important clinical implications for the treatment and management of chronic illness.

## Introduction

Multiple Sclerosis (MS) is a chronic autoimmune disease of the central nervous system that lowers quality of life (Arnett, 2003). While there is currently no cure for MS, medication adherence is important in reducing relapse and disease progression (Goodin et al., 2002). In particular, medication adherence following initial diagnosis of MS has been found to be predictive of disease outcomes as well as future compliance rates (Kleinman et al., 2010). However, given the chronic, uncertain, and life changing nature of MS, patients often experience elevated levels of distress during the early stages of disease (Janssens et al., 2003). Importantly, the first 5–10 years following diagnosis are a time of considerable stress, emotional upheaval, in conjunction with frequent disruptions from changes in physical impairments and increased fatigue (Strober, 2018). Indeed, it is estimated that MS patients are at considerably high risk for psychiatric illness, with rates of lifetime depression approaching 50% (Siegert and Abernethy, 2005). The severe consequences of depression are well-documented, contributing to expensive disruptions in functioning (Kessler et al., 2006), greater risk for physical illness (Blume et al., 2011), and within the context of chronic illness, poor treatment compliance and prognosis (Kalsekar et al., 2006; Moussavi et al., 2007). Therefore, it is important to identify protective factors that may facilitate adherence to MS and related medications.

Research suggests that emotion differentiation (ED; also known as emotional granularity), which refers to the ability to distinguish between emotional states in a fine-grained manner (e.g., fear vs. anger; Barrett et al., 2001), may be important. Individuals who are more adept at differentiating their emotions tend to label their experiences using various terms that describe the presence and intensity of specific emotions (e.g., sadness). Conversely, poorer differentiators are more likely to experience difficulties separating between emotional states and tend to describe their experiences as generally “bad” or “unpleasant” (Kashdan et al., 2015; Smidt and Suvak, 2015). In the literature, ED is commonly assessed using experience-sampling methodology, where individuals repeatedly report their emotional experiences across time. A person-level indicator of ED is derived by calculating the intraclass correlation coefficient (ICC) between emotion ratings across the sampling duration.

Studies accumulated over the past two decades have generally shown that ED, particularly negative ED (NED), is associated with better psychosocial functioning, including lower levels of depression and anxiety (e.g., Demiralp et al., 2012; Seah et al., 2020), and decreased engagement in a wide range of maladaptive behaviors in response to negative emotion across clinical and non-clinical samples (O’Toole et al., 2020; Seah and Coifman, 2021). Indeed, there is compelling theory and evidence suggesting that NED may operate by facilitating adaptive regulation of states of distress (Kashdan et al., 2015). For example, labeling feelings precisely can decrease the intensity of emotion-related arousal (Tabibnia et al., 2008; Kircanski et al., 2012). Moreover, NED has been explicitly tied to adaptive emotion regulatory strategy use (Barrett et al., 2001; Hill and Updegraff, 2012) and has been found to mitigate the association between negative affect (NA) and maladaptive behaviors known to interfere with treatment and predict poorer prognosis, including binge drinking, social avoidance, and non-suicidal self-injury (Kashdan et al., 2010; Zaki et al., 2013; Seah et al., 2020).

While it is now increasingly clear that NED may afford some protection against maladaptive behaviors, less is known about whether it may promote adaptive health behaviors. This appears especially important in the context of chronic illness management, where negative emotional states are known to adversely impact health behaviors such as treatment adherence (Bruce et al., 2010). Results from one prior study by Coifman et al. (2014) found that NED was positively associated with adherence in Beta-Thalassemia, a congenital blood disorder. However, this study only examined attendance at routine screenings and less is known regarding other important indicators of treatment compliance such as daily medication adherence.

Therefore, the present investigation sought to replicate and extend findings by Coifman et al. (2014) in the context of MS by examining NED as a moderator of the association between NA and self-reported medication adherence in daily life. As in previous studies, we utilized sampling methodology via weekly diaries to derive person-level indices of NED, mean NA, and medication adherence rates across a 58-week period. Given its protective benefits, we hypothesized that the effects of NA on medication adherence rates would depend on levels of NED. Specifically, we predicted that NA would be negatively associated with medication adherence at low but not high levels of NED.

## Materials and Methods

### Participants

Data described in the present investigation were part of a larger study investigating emotion processing related to psychological adjustment in MS. Of the total sample, 27 participants (aged 18 and above) were included in the current study as they completed the weekly diary portion that examined emotional experiences and medication adherence in daily life over a period of 1 year. These participants were English-speaking MS patients (aged 18 and above) recruited from a local clinic and/or online advertisements from the Midwestern United States (see Eligibility Criteria and Recruitment). Participants reported a mean (SD) age of 36.11 (8.88), and were mostly female (74.1%), White/Caucasian (81.5%), non-Hispanic (100%), and working full-time or part-time (51.9%). Table 1 describes the characteristics of this sample. All participants provided informed consent prior to the start of data collection.

**TABLE 1:** Characteristics of study participants as a percentage of the sample (*n* = 27).

Characteristics	Study sample
**Sex**	
Female	74.1
Male	25.9
**Race**	
White/Caucasian	81.5
Black/African American	7.4
Other	11.1
**Ethnicity**	
Non-Hispanic or Latino	100
**Employment status**	
Work full-time	33.3
Work part-time	18.5
Retired	3.7
Unemployed	3.7
Unemployed due to disability	33.3
Other	7.4
**Date of multiple sclerosis diagnosis**	
Within past 5 years	92.6
Within past 10 years	7.4
**Diagnostic status**	
Met criteria for any psychological disorder	44.4
Major depressive disorder	7.4
Generalized anxiety disorder	14.8
Social anxiety disorder	14.8
Agoraphobia without panic disorder	11.1
Posttraumatic stress disorder	3.7

#### Eligibility Criteria

The eligibility criteria for participants in the larger study included having a diagnosis of Relapsing –Remitting MS (RRMS), largely because 85% of all MS patients are diagnosed with RRMS at disease onset (McKay et al., 2015). This diagnosis was evaluated by a neurologist according to the revised McDonald Criteria (Polman et al., 2005), which is a diagnostic scheme that provides reliable diagnoses of MS and prevents false positives. The criteria combined magnetic resonance imaging with well-established diagnostic examinations that considered neurological history and examination, and a range of other laboratory tests. Given the larger study’s focus on adjustment following MS diagnosis, participants’ time of MS diagnosis could not exceed 10 years prior, although nearly all (92.6%) were diagnosed within the prior 5 years. In addition, psychiatric history was assessed in a diagnostic interview using the Structured Clinical Interview for Diagnosis of Axis I Disorders in the DSM-IV-TR (SCID-I; First et al., 2002). Participants with a history of psychosis were excluded from the research. See Table 1 for information on individuals who met criteria for current diagnosis of any psychological disorder, as well as specific diagnoses.

#### Recruitment

Participants were primarily recruited from an MS clinic in Northeast Ohio. Patients who met the inclusion criteria were approached by a registered nurse who was certified by the International Organization of Multiple Sclerosis Nurses, a graduate student, or a trained research assistant. Alternatively, participants responded to advertisements posted online. Phone interviews were conducted with individuals who expressed interest in the study to evaluate eligibility and explain the study’s activities. For participants who were recruited online, their diagnoses were verified (with consent) through their neurologist to ensure all eligibility criteria were still met. Sixty-one percent of participants completed the initial data collection at the MS clinic and 39% completed it in a research lab at Kent State University. Ninety-five percent of participants were taking medications to decrease the number of relapses and reduce the progress of the disease and 5%^1^ were not taking any medications for MS relapses. Fifty-five percent of participants who were taking a medication to decrease the number of MS relapses were taking infused/intravenous MS medication (e.g., Tysabri and Lemtrada), 25% were taking self-injection MS medication (e.g., Copaxone, Rebif, and Avonex), 18% were taking oral MS medication (e.g., Tecfidera, Gilenya, and Aubagio), and 2% did not report the delivery type of their medication. In addition to the MS medication that was taken to manage the disease course, other medications (e.g., Gabapentin and Baclofen) were taken to manage MS-related symptoms. The most frequently reported symptoms were pain, such as nerve pain, muscle spasm and spasticity, and bladder problems. The time frame for participant recruitment was from June 2013 to February 2017.

### Procedure

Data collection was completed across an 18-month period. However, data for this study focused exclusively on the 12-month weekly sampling period. During the initial stage (first 3 months), all participants completed two lab sessions that included a diagnostic interview (described above), as well as other tasks, questionnaires, and assessments to determine physical and cognitive functioning in MS. Upon completion of the second session, participants received training to complete a weekly diary for the next 10–12 months (see “Measures” section for more details). During this stage of study participation, they received weekly diary reminders by the research team and returned each diary entry via addressed-stamped envelopes. Note that participants were provided with the option to opt out of this study component. Following completion of the diary, participants returned to the laboratory for two follow-up sessions, where they repeated assessments described in Stage 1. All study procedures were reviewed and approved (Protocol: #13-134) by the university Institutional Review Board before the start of data collection.

### Measures

#### Weekly Diary

Participants were trained by a research assistant to complete weekly diaries to assess their emotional experiences and frequency of medication adherence. Participants completed these diaries for up to a maximum of 58 weeks (approximately 10–12-month period; for 58 diaries). Participants were provided with paper copies of weekly diaries after the second laboratory session. They completed these diaries once a week, typically on the same day each week (e.g., Saturday), and returned these documents to the research team via postal mail. To increase compliance, all participants received weekly reminder phone calls and/or emails from a trained research assistant and packets of paper diaries were mailed monthly to participants. Four participants were excluded from subsequent analyses because they completed less than seven diaries in total, which was less than 1.5 standard deviations from the average number of diaries completed across the sample (Bolger et al., 2003). There were no significant demographic differences (age, sex, race/ethnicity, and employment status) between excluded individuals and the final sample. The final sample (*n* = 23) completed a mean (SD) of 37.26 (12.55) diaries (range: 12–55; compliance: 64%).

##### Momentary Self-Reported Emotions

During each weekly diary, participants were asked to describe their current emotional experience by providing ratings on a 7-point Likert scale ranging from 1 (“none”) to 7 (“strong”). Participants rated the extent to which they felt each of six negative emotion words (*fear*, *sadness*, *guilt*, *distress*, *anger*, *disgust*), which formed the NA scale. Unrelated to the present study, participants also rated how much they felt each of six positive emotion words (*happiness*, *enjoyment*, *affection*, *surprise*, *amusement*, *relief*). As in previous studies on ED, these emotion words were selected to reflect varying levels of activation across negative and positive valence dimensions of contemporary affective circumplex models (e.g., Russell, 1980; Rafaeli et al., 2007). To assess the reliability of the NA scale, we computed values at the between-person (R_KF_) and within-person (R_C_) levels following Cranford et al. (2006). The between-person (R_KF_ = 0.99) and within-person (R_C_ = 0.82) reliability for this sample were good.

##### Mean Negative Affect

An index of mean NA was obtained by calculating a mean score across participants’ ratings of the six negative emotion words from each diary across the sampling duration. The mean (SD) level of mean NA reported in this sample was 1.53 (0.48; range: 1.01–2.79).

##### Negative Emotion Differentiation

As in previous studies, a person-level index of NED is obtained for each participant by computing the average ICC with absolute agreement between negative emotion ratings across all diaries (Kashdan et al., 2015). This ICC index provides a measure of how similarly (i.e., level of agreement) ratings of negative emotion words vary across time. Higher ICC values would suggest similar ratings across negative emotions at any given diary entry, while lower ICCs would suggest more differentiated responses across emotion ratings. One individual had a negative ICC (−0.02) and this value was changed to 0 and included in subsequent analyses (Erbas et al., 2014).^2^ Following existing conventions in ED research, ICC values were reverse scored (i.e., subtracted from 1) so that higher values indicated greater NED for ease of interpretation (Seah and Coifman, 2021). The mean (SD) level of NED reported in this sample was 0.34 (0.25; range: 0.03–1.00), which is comparable to that reported in other clinical and community samples (e.g., Zaki et al., 2013; Seah et al., 2020).

##### Medication Adherence

During each weekly diary, participants were asked to record information about their medications (includes those that treated MS and MS-related symptoms) over the past week and indicated whether they completed (coded as 1) or missed (coded as 0) a dose for each day of the week. As in Coifman et al. (2014), medication adherence rates across the sampling period were derived by calculating the proportion of the total number of completed vs. missed doses across all completed diaries based on the medical records of prescribed medications. The mean (SD) rate of medication adherence reported in this sample was 0.77 (0.31; range: 0–1.00).

### Data Analysis Plan

First, we examined bivariate correlations between primary outcome variables and potential covariates (e.g., age, total number of diaries completed) in the final sample. In addition, a series of one-way ANOVAs were conducted to examine whether there were any demographic differences in medication adherence in terms of sex, race, and employment status. Next, we tested the possible moderation of the association between mean NA and medication adherence by NED using the Hayes PROCESS macro (Model 1) in IBM SPSS (v. 23). This macro runs a series of Ordinary Least Squares regressions with the centered product term representing the interaction of mean NA by NED as a predictor of medication adherence. The estimated effects reported were unstandardized regression coefficients. Statistical significance was set at 0.05.

## Results

### Preliminary Analyses

Results from correlational analyses revealed a significant negative association between mean NA and the total number of completed diaries, *r* = −0.42, *p* = 0.043. Contrary to past research, mean NA was not significantly associated with medication adherence, *r* = −0.22, *p* = 0.323. Similarly, NED was not associated with medication adherence, *r* = 0.14, *p* = 0.529 or mean NA, *r* = −0.06, *p* = 0.778. No other significant correlations were observed. Next, results from one-way ANOVAs revealed no significant differences in medication adherence due to sex, *F*_(1, 21)_ = 0.18, *p* = 0.679, race, *F*_(2, 20)_ = 0.38, *p* = 0.687, or employment status, *F*_(5, 17)_ = 0.97, *p* = 0.462.

### Primary Analyses

**FIGURE 1:**
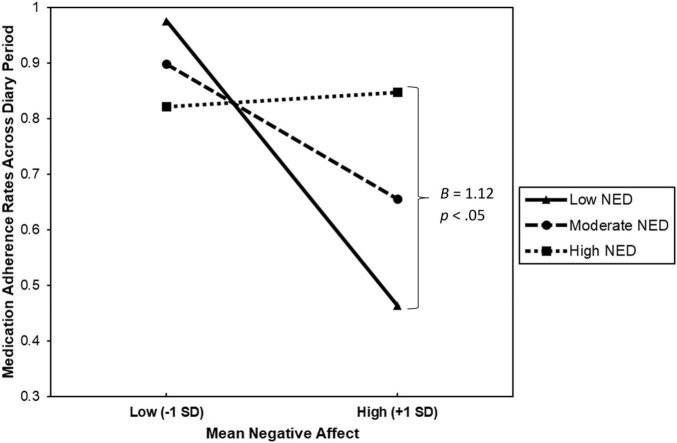
This figure describes the interaction between mean negative affect and negative emotion differentiation (NED) when predicting medication adherence rates across the 58-week diary period among 23 patients with Multiple Sclerosis.

Results from the moderation analyses are presented in Table 2. As hypothesized, we found that NED significantly moderated the relationship between mean NA and medication adherence, *B* = 1.12, Δ*R*^2^ = 0.18, *F*_(1, 19)_ = 4.62, *p* = 0.045, 95% CI [0.03; 2.22], *sr*^2^ = 0.18. This suggests that the impact of mean NA on medication adherence depended on levels of NED. To examine the effects of the interaction, predicted values were plotted for individuals at the mean and ± 1 SD from the mean of NED and mean NA (refer to Figure 1). Follow-up tests of the simple slopes revealed that the association between mean NA and medication adherence under low NED (1 SD below the mean) was significantly different from zero, *b* = −0.53, *p* = 0.029. Therefore, among individuals with low NED, mean NA predicted poorer adherence. However, the association between mean NA and medication adherence for individuals reporting moderate (at the mean; *b* = −0.25, *p* = 0.087) to high (1 SD above the mean; *b* = 0.03, *p* = 0.859) NED was not significantly different from zero. Thus, mean NA did not appear to influence medication adherence rates among individuals with moderate to high NED. Finally, we re-ran our analyses controlling for the total number of completed diaries, as well as diagnostic status (i.e., individuals with vs. without a current diagnosis of a psychological disorder), and the pattern of results remained the same.

**TABLE 2:** Significant Two-way (Mean NA by NED) interaction predicting medication adherence (*n* = 23).

	Predictor	*B*	*SE*	95% CI	*sr* ^2^	*R* ^2^	Δ*R*^2^
Step 1	Mean NA	–0.13	0.14	−0.43 to 0.16	0.04	0.06	
	NED	0.16	0.16	−0.41 to 0.72	0.02		
Step 2	Mean NA	−0.63*	0.27	−1.19 to −0.08	0.23	0.25	0.18*
	NED	–1.49	0.80	−3.17 to 0.20	0.13		
	Mean NA × NED	1.12*	0.52	0.03 to 2.22	0.18		
	*F*_(3, 19)_ = 2.06, *p* = 0.139						

B, unstandardized coefficient; SE, standard error; CI, confidence interval; NA, negative affect; NED, negative emotion differentiation. *p < 0.05.

## Discussion

Our findings build upon past research demonstrating the protective effects of NED. Specifically, the results suggest that beyond its negative association with maladaptive behaviors, NED may also facilitate adaptive health behaviors, such as daily medication adherence. Moreover, these results held even after controlling for important covariates such as number of completed diaries and diagnostic status. Critically, our sample comprised patients who were recently diagnosed with MS, a period of elevated stress for most new patients, and suggests that NED affords protection even in such highly aversive contexts. This appears especially important given that treatment compliance reduces symptom exacerbations in MS, and in so doing, may help to improve patients’ health in addition to psychological well-being and quality of life (Khayyat et al., 2019; Peacock et al., 2021). Finally, these results replicate prior findings associating NED with treatment compliance in patients with a congenital blood disorder (Coifman et al., 2014) and reinforce the importance of considering affective processes in chronic disease management.

Notably, the interaction term between NED and mean NA accounted for 18% of the variance in our model, suggesting that it may be most important to target emotion-related processes in patients showing elevated negative emotion in order to boost medication adherence in MS treatment. This is consistent with a growing body of work that has demonstrated the implicit emotion regulatory benefits of affect labeling, where assigning labels to one’s emotions may facilitate downregulation of NA and psychophysiological indices of distress (e.g., amygdala activation; Torre and Lieberman, 2018). Moreover, past research suggests that ED may counteract maladaptive cognitive-emotional processes such as rumination that often exacerbate NA and increase propensity of maladaptive behavioral engagement (Zaki et al., 2013; Seah et al., 2020). Instead, it is possible that ED may enable one to disengage from difficult experiences (rather than staying “stuck”), and in turn facilitate greater psychological distance and adaptive regulation of negative emotion (Kross and Ayduk, 2017; Seah et al., 2021). Nevertheless, these hypotheses remain preliminary and future research should aim to explicitly test possible underlying mechanisms of ED in relation to adaptive behavioral response to increase our understanding of how it may operate.

The findings from the current study also have clinical implications. Our results suggest that fostering the ability to differentiate and label emotions may have potential to help patients who exhibit elevated negative emotion and distress while experiencing challenges with medication adherence. Indeed, this is a skill commonly addressed in psychotherapy (Greenberg, 2004), such as dialectical behavior therapy (Linehan, 1993), where explicit training in improving patients’ understanding of emotion is associated with reductions in more severe life-threatening behaviors (e.g., suicidal behaviors; DeCou et al., 2019). Given the relatively high incidence and prevalence of common psychiatric disorders in MS (e.g., mood and anxiety disorders; McKay et al., 2018), clinicians may consider identifying patients at-risk for poor adherence and providing referrals as appropriate. While our results are largely preliminary, there is potential for patients to benefit broadly from interventions aimed at developing ED skills nonetheless.

Additionally, that NED, which is derived from more subjective patient-reported outcomes, seem to be able to impact more objective clinical behavioral outcomes such as medication adherence highlights the need to consider psychological factors in medical contexts. Indeed, the effects of psychological factors such as emotion (e.g., fear) on health behaviors (e.g., health screening) have been well-documented in the literature (see review by Consedine and Moskowitz, 2007). This appears particularly important given the chronic and uncertain nature of diseases such as MS, which inflicts significant burden on patients over time. Moreover, compliance with medication regimen is likely impacted by potential negative medication side effects (e.g., pain from injections), as well as other contextual factors (e.g., fear associated with hospital visits; Lynd et al., 2018). Our findings suggest that it may be beneficial for healthcare providers to consider patient-reported outcomes as part of patient care, which may facilitate identification of risk and/or protective factors during the course of treatment.

The results from the present investigation should be considered in light of the following strengths and limitations. First, this was a relatively rare investigation of NED in a high-risk population in relation to an essential and highly adaptive behavioral response to stress (medication adherence during chronic illness). The assessment of both NED and medication adherence was robust and took place over a year-long period of sampling. Most research estimates NED from far shorter periods of time and has almost exclusively targeted maladaptive behavioral responses (Seah and Coifman, 2021). However, our study sample was small and had limited diversity in race/ethnicity and gender. Therefore, our results may not generalize to other racial/ethnic minority groups and non-female-identifying individuals. Despite the small sample, it is important to note that although not a rare disease, MS is still much less common than many other chronic illnesses and thus the total population of MS patients is also small (Evans et al., 2013). As such, our study’s findings remain clinically meaningful and would benefit from future replications in larger and more diverse samples. In the present study, we assessed medication adherence broadly which limited our ability to capture variability in frequency and method of dosing for both MS and MS-related medications. Indeed, there is evidence that certain MS medications (e.g., Lemtrada; Barclay et al., 2019) have considerable variability in dosing and demand for adherence. Moreover, recall bias is possible since adherence was assessed via weekly retrospective self-report. However, it was also important to minimize participant burden particularly given the long duration of sampling (∼12 months). Nevertheless, future research should consider more sensitive measurements of medication adherence. Finally, the correlational nature of our study prevents conclusive interpretations regarding causality. However, accumulating evidence from experimental studies on ED suggests that it plays a causal role in facilitating adaptive behavioral responses in laboratory provocations (Kircanski et al., 2012; Cameron et al., 2013). This remains an important question worthy of future exploration.

Despite its limitations, the current study revealed important findings that highlight the protective benefits of NED in the context of adjustment to chronic stressors such as MS. Specifically, our findings suggest that NED may facilitate enactment of adaptive health behaviors like medication adherence in patients with elevated distress, that may in turn improve patient health. The clinical implications of our results are also apparent, particularly given the uncertain and life changing nature of chronic illnesses like MS. Future replications in larger, more diverse clinical samples would bolster our findings with potential implications for improving the identification of psychological factors that may facilitate greater adaptation to life stress within vulnerable populations.

## Data Availability Statement

The original contributions presented in the study are publicly available. This data can be found here: https://osf.io/h5kyf/.

## Ethics Statement

The studies involving human participants were reviewed and approved by the Kent State University Institutional Review Board. The patients/participants provided their written informed consent to participate in this study.

## Author Contributions

TS and KC analyzed and interpreted the results. TS drafted the initial manuscript. All authors contributed to study design, revisions of the manuscript, and approved the submitted version.

## Conflict of Interest

The authors declare that the research was conducted in the absence of any commercial or financial relationships that could be construed as a potential conflict of interest.

## Publisher’s Note

All claims expressed in this article are solely those of the authors and do not necessarily represent those of their affiliated organizations, or those of the publisher, the editors and the reviewers. Any product that may be evaluated in this article, or claim that may be made by its manufacturer, is not guaranteed or endorsed by the publisher.

## Footnotes

1 These participants were still included in the study because they were also taking other medications for MS-related symptoms.

2 Negative ICC values are common in studies of emotion differentiation. While ICCs should range between 0 and 1 in theory, they can take on small negative values in practice due to sampling error (Cohen et al., 2003). However, rather than excluding these data, Cohen et al. (2003) recommended assigning them a value of 0. Notably, our results remained the same even after excluding the individual with a negative ICC score for negative emotion differentiation.
